# Association of hand-grip strength and non-alcoholic fatty liver disease index in older adults

**DOI:** 10.20463/jenb.2018.0031

**Published:** 2018-12-31

**Authors:** Inhwan Lee, Jinkyung Cho, Jinkook Park, Hyunsik Kang

**Affiliations:** 1 College of Sport Science, Sungkyunkwan University, Seoul Republic of Korea; 2 Department of Exercise Prescription, Dongseo University , Busan Republic of Korea

**Keywords:** older adults, hand-grip strength, non-alcoholic fatty liver disease, steatosis, fibrosis

## Abstract

**[Purpose]:**

This study examined the association of hand-grip strength (HGS) and non-alcoholic fatty liver disease (NAFLD) index in older adults.

**[Methods]:**

This was a cross-sectional study involving 538 older adults with mean age of 74.3±6.4 years. Body composition parameters including height, percent body fat, body mass index (BMI), waist circumference (WC), was determined using body composition analyzer. HGS was assessed using a dynamometer, and NAFLD was diagnosed by the simple NAFLD score (SNS), hepatic steatosis index (HSI), NAFLD fibrosis score (NFS), and fibrosis 4 calculator (FIB-4). Based on relative HGS, subjects were classified as High HGS, Mid HGS, and Low HGS group. Based on SNS, HSI, NFS and FIB-4 score, subjects were classified as High risk and Low risk group. Logistic regression analyses were used to determine the odds ratio (OR) and 95% confidence interval (CI) of HGS levels for having steatosis and fibrosis.

**[Results]:**

There were linear decreases in NAFLD index such as SNS (P<.001), HSI (P<.001), NFS (P=.001), and FIB-4 (P=.041) across incremental HGS levels. Compared to the High HGS group (reference), the Low HGS group had significantly higher ORs of having SNS (OR=4.583, 95% CI=2.608-8.054, P<.001), HSI (OR=11.697, 95% CI=5.261-26.005, P<.001), and NFS (OR=1.709, 95% CI=1.005-2.907, P=.048).

**[Conclusion]:**

The current findings suggest that a lifestyle intervention consisting of a normal weight and physical fitness should be promoted as a preventive means against NAFLD associated with HGS.

## INTRODUCTION

The declining birth rate and prolonged average lifespan have accelerated the global aging phenomenon. In Korea, the proportion of elderly over the age of 65 years among the total population was 13.8% in 2017 and may reach 24.5% by 2030 and 38.1% by 2050^1^. The aging phenomenon is not only related to an increased prevalence of various chronic diseases such as arthritis, stroke, angina, lung diseases, and hypertension but also be closely associated with an increased prevalence of non-alcoholic fatty liver disease (NAFLD) in the elderly^2,3^. According to a previous overseas study, the prevalence of NAFLD at advanced age was approx. 35%, which is higher than that at middle age and attracted increased attention^4^.

NAFLD occurs when the ratio of lipid accumulation in the liver that has not been caused by alcohol intake or virus infection is greater than 5% of the weight of the liver^5^. It is also divided according to the progression stage into simple steatosis, steatohepatitis, and fibrosis, and persistent symptoms may progress towards cirrhosis or liver cancer accompanied by inflammatory reactions and necrosis; thus, a close association with early mortality has been reported^6^. Although the precise mechanism of NAFLD pathogenesis remains unclear, the ‘two-hit’ theory is considered as the most promising mechanism^7^. This theory includes the ‘1^st^-hit’, in which excessive accumulation of triglycerides in the healthy liver leads to simple steatosis, and the ‘2^nd^-hit’, in which oxidative stress and excessive production of inflammatory cytokines drive simple steatosis towards steatohepatitis. The main risk factors of NAFLD are genetic, including age and gender, and clinical, including obesity, type II diabetes, and metabolic syndrome^8^. Various methods for alleviating these factors such as drug therapy, weight loss, and regular exercise, and physical activities have been reported^9,10^. However, in older adults, the side effects of drug therapy and risks of weight loss have highlighted the importance of enhanced physical strength based on regular physical activities and exercise as the most effective alternative for treating NAFLD^11^.

Numerous studies have reported the positive effects of regular physical activities and high physical strength on improving insulin resistance, metabolic syndrome, and obesity^12^. Recently, a close association was reported between the quality of life and sarcopenia in the elderly and NAFLD, which was previously highly emphasized only in children, young adults, and middle-aged^13^. As a result, physical activity levels and high physical strength in older adults have been reported to play a positive role against NAFLD in the elderly by improving insulin resistance, inflammation, and oxidative stress, regardless of the stage of progression^14^. Zelber-Sagi et al.^15^ investigated the association between leisure-time physical activities and NAFLD in 799 adults in Europe and reported that the healthy group exhibited a higher level of weekly physical activities than the NAFLD group, regardless of whether the activity was an aerobic or resistance exercise. Tsunoda et al.^16^ carried out a 4.4-year follow-up investigation of the association between physical activities and NAFLD in 10,146 adults in Asia and reported that the group performing moderate to intensive physical activities more than three times a week showed an approximately 50% lower risk of NAFLD than the group performing moderate to intensive physical activities less than once a week. Furthermore, Cho et al.^17^ investigated the risk of NAFLD according to weekly physical activities in 595 adults in Korea and reported that the group performing more than 1500 MET-minutes of weekly physical activities showed an approximately 5-fold decreased risk of NAFLD. Additionally, Kantartzis et al.^18^ reported that an inversely lower rate of NAFLD was associated with improved physical strength in 50 adults in Europe after the subjects completed a 9-month recommended physical activity program, and Church et al.^19^ reported that a lower prevalence of NAFLD was correlated with higher cardiorespiratory fitness (CRF) based on associations evaluated in 218 adults in the U.S. Based on these findings in Korea and overseas, an important association appears to exist between NAFLD and physical activities and physical strength. In contrast to studies of adults in middle age, few studies have examined the association between NAFLD and muscular strength in older adults, despite its crucial functional impacts. Moreover, most previous studies focused on determining the association between physical strength and simple steatosis, suggesting that studies are needed to evaluate how physical strength is associated with each stage of NAFLD progression from simple steatosis to steatohepatitis to liver fibrosis.

Thus, the present study was conducted to verify the association between hand-grip strength and indicators of non-alcoholic hepatic steatosis and fibrosis in older adults in Korea.

## METHODS

### Subjects

The subjects were 594 older adults over the age of 60 years in Gyeonggi province, without a specific medical condition and who could perform normal daily activities. After excluding 56 subjects with incomplete data because of unmeasured body composition or hand-grip strength or refusing blood collection, 538 subjects (male: 106; female: 432) were selected for data analysis. Additionally, detailed explanations of the purpose and methods of this study were given to the subjects in both written and verbal forms and the subjects provided consent to participate in the study. The characteristics of the study participants are presented in Table 1. This study was approved by the institutional review board of S University (IRB-2015-09-001-002).

**Table 1.** Characteristics of study participants (M ± SD)

Variables		Total (n = 538)	Men (n = 106)	Women (n = 432)	P value
Body composition and health behavior					
Age (years)		74.3 ± 6.4	74.3 ± 6.1	74.3 ± 6.4	0.954
BMI (kg/m^2^)		24.6 ± 3.1	24.0 ± 2.7	24.8 ± 3.1	0.013
WC (cm)		90.3 ± 13.1	92.3 ± 12.8	89.8 ± 13.2	0.075
Body fat (%)		33.7 ± 7.5	24.4 ± 6.5	36.0 ± 5.8	<0.001
Past/current smoking, n (%)		43 (8.0)	41 (38.9)	2 (0.5)	<0.001
Heavy alcohol intake, n (%)		39 (7.2)	39 (36.8)	0 (0.0)	<0.001
Regular exercise, n (%)		281 (71.3)	50 (60.2)	231 (82.2)	0.012
Blood chemistry profiles					
TG (mg/dL)		123.4 ± 67.2	132.6 ± 87.9	121.1 ± 60.9	0.115
TC (mg/dL)		166.2 ± 45.3	161.4 ± 48.2	167.4 ± 44.6	0.225
HDL-C (mg/dL)		48.2 ± 14.6	44.9 ± 13.6	49.0 ± 14.8	0.009
LDL-C (mg/dL)		93.3 ± 36.2	90.0 ± 35.5	94.1 ± 36.4	0.292
FBG (mg/dL)		115.3 ± 24.3	116.3 ± 27.8	115.1 ± 23.4	0.656
AST (U/L)		21.1 ± 10.1	20.7 ± 9.5	21.2 ± 10.3	0.671
ALT (U/L)		15.3 ± 7.8	16.4 ± 8.7	15.0 ± 7.6	0.095
AST/ALT ratio		1.5 ± 0.6	1.4 ± 0.5	1.5 ± 0.6	0.039
Platelet (109/L)		724.0 ± 448.8	701.5 ± 464.3	729.7 ± 445.5	0.590
Albumin (g/dL)		4.8 ± 0.3	4.8 ± 0.3	4.8 ± 0.3	0.954
Comorbidities					
Dyslipidemia, n (%)		262 (49.0)	41 (38.7)	221 (51.5)	0.018
Diabetes, n (%)		117 (21.7)	26 (24.5)	91 (21.1)	0.439
NAFLD definition					
Steatosis	SNS	8.9 ± 2.7	8.3 ± 2.5	9.0 ± 2.7	0.027
	HSI	32.7 ± 4.4	31.0 ± 4.2	33.1 ± 4.3	<0.001
Fibrosis	NFS	-3.7 ± 6.0	-3.6 ± 6.2	-3.7 ± 6.0	0.920
	FIB-4	1.13 ± 1.44	1.10 ± 1.20	1.14 ± 1.50	0.748
Hand-grip strength					
Hand-grip strength (kg)		21.8 ± 7.1	30.8 ± 6.5	18.8 ± 5.0	<0.001
Relative hand-grip strength (kg/kg)		0.36 ± 0.11	0.47 ± 0.10	0.33 ± 0.09	<0.001

BMI: body mass index, WC: waist circumference, TG: triglyceride, TC: total cholesterol, HDL-C: high density lipoprotein cholesterol, LDL-C: low density lipoprotein cholesterol, FBG: fasting blood glucose, AST: aspartate aminotransferase, ALT: alanine aminotransferase, NAFLD: non-alcoholic fatty liver disease, SNS: simple NAFLD score, HSI: hepatic steatosis index, NFS: NAFLD fibrosis score, FIB-4: fibrosis 4 calculator

### Measured variables and methods of analysis

#### Body composition parameters

Height was measured using an automatic measuring device (DS-102, Jenix, Seoul, Korea) and body weight and percent body fat were measured with an X-Scan Body Composition Analyzer (Jawon Medical, Daejeon, Korea). Body mass index (BMI) was calculated based on weight (kg)/height (m^2^). To determine the waist circumference, trained investigators used an anthropometric tape to measure at the midpoint between the lower border of the rib cage and iliac crest from the left side of the subject.

#### Health behavior parameters

Health behavior parameters included smoking, frequency of alcohol intake per week, and regular exercise. For smoking, the subjects who smoked more than five packs of cigarettes throughout his or her life were categorized as past/current smoking^20^. For the frequency of alcohol intake per week, the subjects were categorized as heavy alcohol intake if the frequency was more than twice per week^21^. The definition of regular exercise was based on the level and frequency of weekly physical activities based on the Korean version of the International Physical Activity Questionnaire Short Form^22,23^, and the subjects who did not meet the criteria of more than 30 min of moderate to intensive physical activity twice per week were categorized as *no regular exercise.*

#### Dyslipidemia and diabetes

Dyslipidemia was defined as a case diagnosed by a physician or based on one of the following: guidelines for the management of dyslipidemia^24^; triglyceride (TG) ≥150 mg/dL, total cholesterol (TC) ≥200 mg/dL, high-density lipoprotein cholesterol (HDL-C) ≤40 mg/dL, or low-density lipoprotein cholesterol (LDL-C) ≥130 mg/dL.

Diabetes was defined as a case diagnosed by a physician or based on the criteria of fasting blood glucose (FBG) ≥126 mg/dL^25^. 

#### Blood collection and analysis of blood parameters

For blood collection, 10 mL of cubital venous blood was collected from the subjects using a disposable syringe after over 8 h of fasting. From the collected blood, 7 mL was placed in a serum tube and centrifuged for 10 min in a 4°C (MF-300, Hanil, Incheon, Korea) at 3,000 RPM and then stored at –80°C deep until analysis; the remaining 3 mL was placed in a heparinized/EDTA tube for platelet analysis using Sysmex-XN1000 (Sysmex, Kobe, Japan) based on the principle of bioimpedance. The stored serum was analyzed for TG, TC, HDL-C, FBG, and albumin using AU680 reagents (Beckman Coulter, Brea, CA, USA) and an enzymatic and colorimetric assay. Additionally, aspartate aminotransferase (AST) and alanine aminotransferase (ALT) were analyzed using the modified IFCC UV kinetic method and Cobas 8000 reagents (Roche, Basel, Switzerland).

#### Definition of NAFLD (hepatic steatosis and fibrosis)

NAFLD was defined as steatosis and fibrosis in the liver. To screen for hepatic steatosis, the simple NAFLD score (SNS) and hepatic steatosis index (HSI), which have proven reliability and validity in Korean subjects^23,26^, were used. The parameters of SNS were age, BMI, waist circumference, diabetes or dyslipidemia, alcohol intake, regular exercise, and menopause, which were used to categorize subjects with a score of ≥8 as NAFLD high risk. The parameters of HSI were AST, ALT, BMI, gender, and diabetes, which were used to categorize subjects with a score >36 as NAFLD high risk, those with a score <30 as normal, and those with a score of 36–30 as moderate. To screen for hepatic fibrosis, the NAFLD fibrosis score (NFS) and fibrosis 4 calculator (FIB-4), which have proven reliability and validity in Korean subjects^27,28^, were used. The parameters of NFS were age, FBG, BMI, platelet, albumin, and AST/ALT ratio, which were used to categorize subjects with a score <-1.455 as normal, those with a score >0.675 as high risk, and those with a score ≤-1.455 and ≤0.675 as moderate risk. The parameters of FIB-4 were age, platelet, AST, and ALT, which were used to categorize subjects with a score >2.67 as high risk, those with a score <1.30 as low risk, and those with a score ≥1.30 and ≤2.67 as moderate.

#### Assessment of hand-grip strength

Hand-grip strength (HGS) was assessed using a dynamometer (TKK-5401, Takei, Niigata, Japan) while the subject was standing straight, and measurements were conducted twice from the dominant hand of the subject per kg. The resting time between the two measurements was 1 min. The maximum value between the two measured values was used, and the relative HGS was calculated based on HGS (kg)/weight (kg) as describe previously^29^.

### Data analysis

All continuous data obtained in the present study were expressed as means and standard deviations, and categorical variables were expressed as ratios per group. Based on relative HGS, the subjects were classified as Low HGS (for the lower 33% in both genders); Mid HGS (for the middle 33%); or High HGS (for the upper 33%). The contrasts polynomial of one-way analysis of variance was used to test for linear trends among the measured parameters according to the level of relative HGS. Additionally, the χ^2^ test was used to examine the ratios among categorical variables based on the level of relative HGS and gender. Next, the subjects were classified into two groups according to non-alcoholic hepatic steatosis based on SNS and HSI, and into further two groups according to fibrosis based on NFS and FIB-4. Next, the odds ratio (OR) of the level of relative HGS was estimated for non-alcoholic hepatic steatosis and fibrosis. All statistical analyses were conducted using SPSS version 23.0 software (SPSS, Inc., Chicago, IL, USA)), and the significance level (α) for hypothesis testing was set to 0.05.

## RESULTS

### Comparison of measured parameters according to HGS levels

Table 2 presents the result of comparing the measured parameters among groups according to the levels of HGS. The results show a significant linear decrease in age (*P* < 0.001), BMI (*P* < 0.001), waist circumference (*P* < 0.001), body fat (*P* < 0.001), dyslipidemia (*P* = 0.011), diabetes (*P* = 0.029), TG (*P* = 0.001), and FBG (*P* = 0.011) with increasing HGS levels, whereas a significant linear increase was observed for HDL-C (*P* = 0.001) and platelets (*P* = 0.005). Additionally, with increasing HGS levels, the SNS (*P* < 0.001) and HSI (*P* < 0.001) which indicate hepatic steatosis related to NAFLD and the NFS (*P* = 0.001) and FIB-4 (*P *= 0.041) which indicate hepatic fibrosis showed significant decreases.

**Table 2.** Measured parameters according to hand-grip strength (HGS) levels (M ± SD)

Variables		Low HGS(n = 179)	Mid HGS (n = 180)	High HGS (n = 179)	P for linear trends
Women, n (%)		144 (33.3)	144 (33.3)	144 (33.3)	1.000
Body composition and health behavior					
Age (years)		76.1 ± 5.8	74.2 ± 5.9	72.6 ± 6.8	<0.001
BMI (kg/m^2^)		26.0 ± 3.2	24.9 ± 2.8	23.1 ± 2.6	<0.001
WC (cm)		94.2 ± 13.5	90.5 ± 12.1	86.1 ± 12.7	<0.001
Body fat (%)		36.3 ± 7.3	34.0 ± 7.0	30.8 ± 7.3	<0.001
Past/current smoking, n (%)		20 (11.2)	11 (6.1)	12 (6.7)	0.155
Heavy alcohol intake, n (%)		11 (6.1)	14 (7.8)	14 (7.8)	0.541
Regular exercise, n (%)		93 (68.9)	92 (72.4)	96 (72.7)	0.487
Blood chemistry profiles					
TG (mg/dL)		135.1 ± 80.9	125.6 ± 65.3	109.6 ± 49.4	0.001
TC (mg/dL)		166.1 ± 48.4	166.3 ± 45.8	166.2 ± 42.7	0.999
HDL-C (mg/dL)		45.7 ± 13.2	47.8 ± 14.5	51.2 ± 15.7	0.001
LDL-C (mg/dL)		93.4 ± 38.9	93.4 ± 34.6	93.1 ± 35.2	0.994
FBG (mg/dL)		118.3 ± 25.3	116.7 ± 27.4	110.9 ± 19.0	0.011
AST (U/L)		21.8 ± 10.8	21.7 ± 12.5	19.8 ± 5.5	0.098
ALT (U/L)		15.7 ± 8.9	15.9 ± 8.7	14.3 ± 5.4	0.107
AST/ALT ratio		1.6 ± 0.7	1.5 ± 0.7	1.5 ± 0.4	0.512
Platelet (10^9^/L)		694.8 ± 432.8	661.4 ± 426.2	819.5 ± 474.3	0.005
Albumin (g/dL)		4.8 ± 0.3	4.8 ± 0.3	4.8 ± 0.3	0.602
Comorbidities					
Dyslipidemia, n (%)		98 (54.7)	90 (50.8)	74 (41.3)	.011
Diabetes, n (%)		46 (25.7)	44 (24.4)	27 (15.1)	.029
NAFLD definition					
Steatosis	SNS	10.0 ± 2.7	9.0 ± 2.5	7.7 ± 2.4	<0.001
	HSI	34.1 ± 4.6	33.1 ± 4.4	30.8 ± 3.4	<0.001
Fibrosis	NFS	-3.0 ± 5.8	-2.9 ± 5.7	-5.2 ± 6.3	0.001
	FIB-4	1.15 ± 1.29	1.33 ± 1.75	0.92 ± 1.18	0.041
Hand-grip strength					
Hand-grip strength (kg)		16.5 ± 5.5	21.7 ± 6.2	25.3 ± 6.7	<0.001
Relative hand-grip strength (kg/kg)		0.26 ± 0.06	0.36 ± 0.06	0.46 ± 0.10	<0.001

HGS: hand-grip strength, BMI: body mass index, WC: waist circumference, TG: triglyceride, TC: total cholesterol, HDL-C: high density lipoprotein cholesterol, LDL-C: low density lipoprotein cholesterol, FBG: fasting blood glucose, AST: aspartate aminotransferase, ALT: alanine aminotransferase, NAFLD: non-alcoholic fatty liver disease, SNS: simple NAFLD score, HSI: hepatic steatosis index, NFS: NAFLD fibrosis score, FIB-4: fibrosis 4 calculator

### OR for non-alcoholic hepatic steatosis and fibrosis according to HGS levels

Table 3 shows the OR estimates for non-alcoholic hepatic steatosis and fibrosis according to HGS levels. For the SNS, an indicator of hepatic steatosis, the ORs of Mid HGS (OR = 2.474, 95% CI = 1.474–4.152, *P* = 0.001) and Low HGS (OR = 4.583, 95% CI = 2.608–8.054, *P* < 0.001) were significantly higher than the OR of High HGS. Similarly, for the HSI, the ORs of Mid HGS (OR = 7.590, 95% CI = 3.430–16.792, *P* < 0.001) and Low HGS (OR = 11.697, 95% CI = 5.261–26.005, *P* < 0.001) were significantly higher than those of High HGS. Even after correcting the values for age and gender, the ORs of Mid HGS (OR = 2.406, 95% CI = 1.425–4.060, *P* = 0.001) and Low HGS (OR = 4.291, 95% CI = 2.405–7.655, *P* < 0.001) were significantly higher than those of High HGS for the SNS; the ORs of Mid HGS (OR = 9.890, 95% CI = 4.197–23.307, *P* < 0.001) and Low HGS (OR = 16.484, 95% CI = 6.837–39.745, *P* < 0.001) were significantly higher than those of High HGS for the HSI. When the values were corrected for percent body fat and smoking in addition to age and gender, Mid HGS (OR = 4.618, 95% CI = 1.464–14.568, *P* = 0.009) showed significantly higher ORs than High HGS for the HSI, while no significant differences were observed for the SNS.

**Table 3.** Odds ratios of having increased steatosis and fibrosis risks according to hand-grip strength (HGS) levels

	Crude (95% CI)	*P*	Model 1 (95% CI)	*P*	Model 2 (95% CI)	*P*
Increased steatosis risk						
SNS						
High HGS	1 (ref)		1 (ref)		1 (ref)	
Mid HGS	2.474 (1.474–4.152)	0.001	2.406 (1.425–4.060)	0.001	1.301 (0.699–2.421)	0.406
Low HGS	4.583 (2.608–8.054)	<0.001	4.291 (2.405–7.655)	<0.001	1.615 (0.810–3.221)	0.174
HSI						
High HGS	1 (ref)		1 (ref)		1 (ref)	
Mid HGS	7.590 (3.430–16.792)	<0.001	9.890 (4.197–23.307)	<0.001	4.618 (1.464–14.568)	0.009
Low HGS	11.697 (5.261–26.005)	<0.001	16.484 (6.837–39.745)	<0.001	2.985 (0.877–10.156)	0.080
Increased fibrosis risk						
NFS						
High HGS	1 (ref)		1 (ref)		1 (ref)	
Mid HGS	1.841 (1.087–3.118)	0.023	1.714 (1.006–2.921)	0.048	1.367 (0.784–2.385)	0.270
Low HGS	1.709 (1.005–2.907)	0.048	1.444 (0.835–2.498)	0.189	1.003 (0.549–1.836)	0.991
FIB-4						
High HGS	1 (ref)		1 (ref)		1 (ref)	
Mid HGS	2.067 (0.983–4.343)	0.055	1.791 (0.839–3.824)	0.132	1.387 (0.622–3.097)	0.424
Low HGS	1.617 (0.746–3.505)	0.223	1.165 (0.524–2.592)	0.708	0.881 (0.366–2.122)	0.777

Crude was non-adjusted

Model 1 was adjusted for age and sex

Model 2 was adjusted for age, sex, percent body fat, and smoking

HGS: hand-grip strength, CI: confidence interval; SNS: simple NAFLD score, HSI: hepatic steatosis index, NFS: NAFLD fibrosis score, FIB-4: fibrosis 4 calculator

For NFS, an indicator of hepatic fibrosis, significantly higher ORs were observed for Mid HGS (OR = 1.841, 95% CI = 1.087–3.118, *P* = 0.023) and Low HGS (OR = 1.709, 95% CI = 1.005–2.907, *P* = 0.048) than for High HGS. After correcting the values for age and gender, the OR of Mid HGS (OR = 1.714, 95% CI = 1.006–2.921, *P* = 0.048) was significantly higher than that of High HGS, and when the values were additionally corrected for percent body fat and smoking, no significant differences were observed. For FIB-4, the OR according to HGS levels did not show significant differences.

## DISCUSSION

This study aimed to examine the association between HGS levels and indicators of non-alcoholic hepatic steatosis and fibrosis in 538 older adults. Relative HGS was classified into three groups based on the HGS and weight of the subject, and the scores for steatosis and fibrosis were compared. The results showed that the group with a higher HGS displayed a significant decrease in scores for steatosis and fibrosis. When the subjects were categorized into the low-risk and high-risk groups for the steatosis indicators SNS and HSI and fibrosis indicators NFS and FIB-4 based on a previous study, the estimated ORs of HGS levels suggested that when HGS levels were lower, the ORs of having non-alcoholic hepatic steatosis or fibrosis were higher.

Because of the rapid increase in the global aging society, problems related to musculoskeletal and chronic disorders in older adults have increased, and the prevalence of NAFLD was shown to be higher in advanced age than in middle age, which has recently attracted increased attention^3^. NAFLD includes all aspects of the progression of fatty liver disease not caused by alcohol intake or virus infection. When the symptoms persist, the condition is closely associated with liver cancer and early mortality^6^. Currently available methods for diagnosing NAFLD include liver biopsy, radiological diagnosis, and hematological tests. However, these methods are invasive and costly. Thus, recent studies have been conducted to develop methods of diagnosing non-alcoholic hepatic steatosis and fibrosis using hematological parameters^30^. The present study also analyzed the blood parameters and health behavior of the subjects and examined separate groups of subjects with respect to the indicators of steatosis and fibrosis. The results indicated high-risk groups for steatosis as 50.5% SNS and 18.4% HSI and for fibrosis as 27.3% NFS and 11.4% FIB-4, which are similar to the values observed in previous overseas studies^4,31^.

The most well-known risk factors of NAFLD are obesity, insulin resistance, and type II diabetes, and numerous studies have investigated effective ways to improve such metabolic disorders^8^. As a result, regular physical activities and exercise were reported as the most effective methods for preventing and treating NAFLD, as they lower obesity, enhance insulin sensitivity, and improve blood glucose metabolism^12,32^. A high level of physical strength is also known to have positive effects on the prevention of NAFLD^11^. In line with this, we classified the relative HGS into three groups based on the HGS and body weight of older adults and compared the scores for steatosis and fibrosis. The result showed that the group with a higher HGS level displayed a significant linear decrease in the scores for non-alcoholic hepatic steatosis and fibrosis. The findings agree with those of Krasnoff et al.^33^ who evaluated 37 adults in the U.S. to determine the association between NAFLD and CRF and reported a lower CRF in the group diagnosed with non-alcoholic hepatic steatosis or steatohepatitis. Additionally, Nagano et al.^34^ evaluated 84 adults in Japan to determine the association between CRF and NAFLD enzymes and found that a higher CRF led to lower levels of AST and ALT. The findings of our study and previous studies suggest that regular physical activities and high physical strength in older adults have positive effects on improving the main causes of NAFLD: insulin resistance, metabolic syndrome, and obesity^14^, while sarcopenia may be an independent predictive parameter of NAFLD^15^.

Furthermore, when the ORs for non-alcoholic hepatic steatosis or fibrosis were estimated based on the categorized HGS levels, the OR of the Low HGS group was significantly higher than that of the High HGS group with respect to the SNS, HSI, and NFS. However, when the values were corrected for age, gender, percent body fat, and smoking, only the HSI showed a higher OR in the Mid HGS group than in the High HGS group, while the other indicators showed no significant differences. The findings agree with those of Meng et al.^29^ who examined the association between relative HGS and NAFLD in 20,957 Chinese adults and reported that a higher HGS led to a significantly lower OR for NAFLD. Additionally, Peng et al.^13^ examined the association between NAFLD and muscle mass and gait velocity in 2,551 older adults in the U.S. and found that as the severity of NAFLD increased, muscle mass and gait velocity decreased. A high level of physical strength may be a crucial parameter for improving NAFLD, as it prevents sarcopenia and obesity while reducing the risk of chronic diseases in older adults^36,37^. Nonetheless, when the values were corrected for percent body fat and smoking, the OR for NAFLD according to HGS levels was shown to be only partially significant, indicating that health behaviors reflected by percent body fat and smoking are important additional factors to consider in the association between NAFLD and physical strength in older adults. Despite this, most previous studies investigated the association between physical strength and simple steatosis. In contrast, the current multilateral study was carried out to determine the association between the HGS and NAFLD including simple steatosis and hepatic fibrosis.

## CONCLUSION

In summary, the relative HGS based on the HGS and weight of older adults are inversely related to non-alcoholic hepatic steatosis and fibrosis. Thus, muscular strength in older adults is a potential predictive parameter for NAFLD. The limitations of the study include the difficulty verifying the cause-effect relationships as this was a cross-sectional study; the fact that hematological parameters were used to diagnose NAFLD; and the lack of nutrition survey. Further studies using a prospective design and more precise methods for diagnosing NAFLD are needed.
